# Disturbances in time perception in patients with obsessive-compulsive disorder

**DOI:** 10.1007/s00406-026-02260-8

**Published:** 2026-04-29

**Authors:** Paraskevi Mavrogiorgou, Homan Amanpour, Thomas Jedamzik, Georg Juckel

**Affiliations:** Department of Psychiatry, Psychotherapy, and Preventive Medicine, LWL University Hospital, Ruhr University Bochum, Alexandrinenstr. 1, 44791 Bochum, Germany

**Keywords:** Obsessive-compulsive disorder, Time perception, Time processing, Compulsive slowness

## Abstract

**Background:**

The experience of time and the temporal order of external and internal events is essential for humans. Impairments in the experience of time have long been discussed in relation to main psychiatric disorders, but rarely in relation to obsessive-compulsive disorder. This pilot study therefore investigated impairments in the experience of time in terms of time perception and time awareness in patients with obsessive-compulsive disorder, as well as possible neurobiological correlates.

**Method:**

Twenty patients (11 men and 9 women, mean age 43.3, SD = 14.3) with an ICD-10 diagnosis of obsessive-compulsive disorder (F42.X) and 20 healthy subjects matched by age and gender (13 men and 7 women, mean age 40.2, SD = 15.4) were examined using time experience questionnaires, time estimation tasks, and flicker fusion frequency.

**Results:**

Significant differences were found in the experience of time between patients with obsessive-compulsive disorder and healthy control subjects. The patient group scored lower on time knowledge, time perception, and time management. It is worth mentioning the positive significant correlation (*r* = 0.641; *p* = 0.002) between the severity of compulsive behavior and impaired time management.

**Discussion:**

Disturbances in the experience of time appear to play an important role in psychiatric disorders, also in OCD. It is suggested that the process changes in the experience of time should be given greater consideration in current psychopathological findings.

## Introduction

With a lifetime prevalence of approximately 1–3%, obsessive-compulsive disorders (OCD) are among the most common mental illnesses in both adults and children and adolescents [1]. Phenomenologically, obsessive-compulsive disorder is characterized by the occurrence of obsessive thoughts and/or compulsive behaviors, which are associated with a high level of suffering for those affected (and often their relatives) and with a significant impairment of everyday life. The phenomenological heterogeneity and variability of OCD indicates that compulsive symptoms may be based on multifactorial and pathogenetically heterogeneous disease processes [2]. This, in turn, seems to explain the sometimes contradictory research results in connection with the following and currently predominant etiopathogenetic models of obsessive-compulsive disorder. The neurotransmitter model or neurochemical hypothesis of obsessive-compulsive disorder essentially postulates a serotonergic dysfunction in the sense of a central serotonin dysregulation. This is also supported by findings of a strong loudness dependence of acoustically evoked potentials (LDAEP) as an indicator of low central serotonergic transmission [3]. The neuroanatomical model of the pathogenesis of obsessive-compulsive disorder postulates a disturbance in the anatomical connection loops, particularly between the frontal cortex, the thalamus, and the basal ganglia [1]. The specific pathogenetic role of the basal ganglia, which are essential for the regulation of motor function and movement, as well as the genetic findings regarding glutamatergic abnormalities are still not fully understood in OCD.

In this context, the phenomenon of “compulsive slowness” (Primary Obsessive Slowness = POS) should be mentioned. Compulsive slowness [4] is considered a typical phenomenon of obsessive-compulsive disorder in the sense of motor slowing and consecutive restriction of everyday activities such as washing, dressing, etc [5, 6]. However, the spectrum of motor disorders includes both gross motor disorders, abnormal involuntary movements, and fine motor coordination deficits, which are referred to as “soft neurological signs” [7]. This results in, among other things, a loss of motor fluidity, hesitation in initiating movements of the extremities, but also complex repetitive movement patterns such as tics [6, 8, 9]. It is assumed that the complex motor deficits cause difficulties in initiating purposeful actions, which in turn manifests itself as primary “compulsive slowness” [10]. This is distinguished from “secondary compulsive slowness,” which results from repetitive compulsive actions and repetitive compulsive rituals [11]. Recent publications, mostly in the form of case studies [8, 12, 13], which viewed slowness in the cases presented as a consequence of complex motor changes in the context of an early manifestation of obsessive-compulsive disorder, call for a thorough neuromotor examination of the affected patients in addition to the abolition of the distinction between primary and secondary compulsive slowness. Furthermore, the extent to which the performance of repetitive, stereotypical compulsive actions and rituals (which often manifest themselves as secondary slowness in everyday life) is related to an altered experience of time in patients with obsessive-compulsive disorder needs to be discussed. This is surprising, especially since an early case study [14] emphatically describes the patient’s “time-related compulsion to record” [14] and her cognitively compulsive fixation on time. Although a connection between altered perception of time or experience of time and obsessive-compulsive disorder was suggested early on, this connection has been completely neglected in the field of research on obsessive-compulsive disorders. Only a more recent publication [15], based in part on the famous work of Gebsattel, attempts to approach the topic of time perception and obsessive-compulsive disorders presenting a 28-year-old female patient with pronounced and long-lasting control rituals and clear characteristics of compulsive slowness. Interestingly, the experience of time in this case is not linear but circular (“endless loops”).

Time processing is generally considered to be the umbrella term for all time processing processes [16]. It is important to distinguish between the objective dimensions of time (circadian rhythms, internal and external timekeepers) and the subjective dimensions of time experience (knowledge of time, experience of time, use of time, and perception of time). Knowledge of time encompasses the understanding of basic temporal relationships (before, after, past, present, and future). *Time experience* describes the individual’s perception of the passage/flow of time (drawn out, standing still). *Time management* describes how we deal with our own lifetime and *time perception* (also defined as “temporal processing”) [17, 18] and encompasses the ability to estimate the duration of a process and the chronological order of events. According to current knowledge, disturbances in the objective experience of time in the sense of knowledge of basic temporal relations are usually only found in neuropsychiatric diseases such as dementia, encephalitis, brain damage and so on, while changes in the subjective perception of time have been found in schizophrenic patients, but less so in depressive patients [16–18]. In contrast, subjective experience of time appears to be disturbed in many psychiatric disorders [19]. However, it remains unclear how nosology-specific these are and, in particular, how subjective experience of time as a psychopathological abnormality can best be scientifically examined without resorting solely to the phenomenological level of individual cases.

The aim of our exploratory cross-sectional study was therefore to record differences in the experience of time in patients with OCD and in comparison to healthy subjects under standardized conditions and using a combination of instrumental measurement methods to collect neurobiological parameters.

## Method

### Patients and subjects

The study examined 20 patients (11 men and 9 women, mean age 43.3, SD = 14.3) diagnosed with OCD (F42.X) without any further diagnosed psychiatric comorbidity according to ICD-10 and 20 healthy subjects (13 men and 7 women, mean age 40.2 (SD = 15.4)). A more detailed sociodemographic characterization of the entire study group can be found in Table 1. With the exception of completed vocational training in favor of OCD patients, there were no significant group differences in the sociodemographic characteristics. There was no significant difference concerning the cumulative time in education and the MWT-B did not differ.

**Table 1:** Sociodemographic and clinical characteristics of the study groups

Trait	OCD Pat. *N* = 20	Healthy subjects *N* = 20	*p*-value*
Age, M (SD), years	43.3 (14.3)	40.2 (15.4)	n. s.
Gender			n. s.
Female n,	9 (45%)	7	
Male n,	11 (55%)	13 (65%)	
Family and relationship status			n. s.
Single, n	11 (55%)	16 (80%)	
Married, n	6 (30%)	4 (20%)	
Divorced, n	2 (10%)	0	
Widowed	1 (5%)	0	
Currently without a partner	8	11 (55%)	n. s.
In a relationship, n	12 (60%)	9 (45%)	
Children	7 (35%)	4	n. s.
No children	13 (65%)	16	
Level of education			
High school diploma, n	12 (60%)	15 (75%)	n. s.
Technical college entrance qualification, n	6 (30%) 0	2 (10%) 2	
Secondary school diploma, n	2	1 (5%)	
Occupational status			
Completed vocational training	19 (95%)	14 (70%)	*p* = 0.037
None	1 (5%)	6	
Currently employed, n	15 (75%)	10	
Not currently employed, n	5 (25%)	10	n. s.
Unemployed	2	2	
School pupils/students	2	6	
Retired	1	2	
PSP	67.8 (15.1)	92.5 (11.6)	*p* < 0.001
CGI	4 (1.1)	1 (0)	p *≤* 0.001
BDI-II	14.7 (9.6)	4.7 (6.3)	p *≤* 0.001
STAI-I	41.5 (9.9)	30.9 (6.4)	p *≤* 0.001
STAI-II	50.2 (10.6)	32.5 (8.1)	p *≤* 0.001
MWT-B	111.1 (15.0)	113.2 (13.0)	*p* = 0.647; n. s.

*M* mean, *SD* standard deviation, *n* number, *BDI-II* Beck Depression Inventory-II,; *STAI* State and Trait Anxiety Inventory, *n. s.* not significant

*x^2^ test/Mann-Whitney U test

A positive family history of mental illness was found significantly more frequently (*p* = 0.013) in the 20 OCD patients examined here. The majority (90%, *n* = 18) of the patients examined had an ICD-10 diagnosis of obsessive-compulsive disorder with mixed obsessive thoughts and compulsive actions (F42.2), one patient had F42.0 (predominantly obsessive thoughts) and another patient had F42.1 (predominantly compulsive actions).

These were also clinically characterized by an average duration of illness of 21.0 (SD = 12.0) years, with a range between 4 and 47 years. The average age at onset of the illness was 22.3 (SD = 11.0) years.

The inclusion criteria for both patients and healthy subjects were defined as age between 18 and 70 years, normal estimated premorbid verbal IQ and sufficient German language skills. The exclusion criteria for both groups included participation in another clinical study no longer than 1 month prior, lack of consent to participate in the study, the presence of addiction (illegal drugs such as opioids, amphetamines, and so on), personality disorder, and organic brain disorders. In addition, patients with severe neurological or unstable internal medical conditions, acute suicidality or behavior that posed a danger to others were excluded from participating in the study. Mentally healthy subjects with severe somatic illness were also excluded. Existing medication taken by the patients was not an exclusion criterion for participation in the study.

At the time of the examination, all (OCD) patients (*n* = 20, 100%) were taking antidepressant medication, predominantly from the SSRI class (primarily sertraline in doses of 100–300 mg/day (*n* = 6), escitalopram 20 mg/day (*n* = 3) or citalopram 10 to 20 mg/day (*n* = 2), paroxetine 40 mg/day (*n* = 1)), followed by the SNRI group (primarily venlafaxine between 75 and 300 mg/day (*n* = 5) and duloxetine 120 mg/day (*n* = 1)). In addition, 6 of the 20 patients (30%) received additional medication in the form of neuroleptic augmentation consisting of aripiprazole (15 mg/day in *n* = 2) or quetiapine (50–100 mg/day in *n* = 2) or promethazine (10–50 mg/day as needed in *n* = 2). Only 2 patients were treated with clomipramine (75–300 mg/day). Outpatient psychotherapy was reported in the medical history of 12 of the 20 patients (= 60%).

A positive vote was obtained from the Ethics Committee of the Medical Faculty of the Ruhr University Bochum (No. 23-7802). Consent to participate in the study was obtained in accordance with the Helsinki and ICH-GCP declarations.

### Psychometrics

Biographical and clinical data were collected from all study participants in a one-time semi-structured interview using internal clinic anamnesis forms and the Mini-PLUS interview guide. Patients were recruited from the clinic’s long-standing special consultation hours for obsessive-compulsive disorders. The healthy subjects were recruited from the wider circle of acquaintances of the project staff. A number of questionnaires were also used for psychometric testing, such as the Beck Depression Inventory (BDI-II) to assess the severity of depression. Anxiety was assessed using the STAI (State-Trait Anxiety Inventory). Assessing the general clinical impairment caused by the symptoms, the Clinical Global Impressions severity and improvement scores (CGI) were used, and the Personal and Social Performance Scale (PSP) was used to assess the level of psychosocial functioning. Verbal intelligence was determined using the Multiple Choice Vocabulary Test (MWT-B). The established Y-BOCS checklist and Y-BOCS severity scale were used to record compulsive symptoms, while functional motor impairments were assessed using the Function Questionnaire Motoric (FFB-MOT) [20]. The psychometric findings of patients with obsessive-compulsive disorder and the healthy comparison group, as assessed using the questionnaires described above, are presented in Table 1.

With an average score of 4 (SD = 1) on the CGI and a mean score of 67.8 9 (SD = 15.1) on the PSP scale, patients with obsessive-compulsive disorder can be classified as clearly ill and with pronounced functional impairments in at least two of the four psychosocial areas (work and personal relationships).

The patients were moderately to severely impaired in terms of both depressive symptoms (BDI-II) and anxiety (STAII/II) and significantly more impaired than the healthy subjects. The verbal intelligence scores (MWT-B) did not differ significantly from those of the healthy subjects and were within the average normal range for both groups.

The severity of obsessive thoughts (8.5 (SD = 3.1)) and compulsive behaviors (8.7 (SD = 3.8)) was classified as mild to moderate based on the Y-BOCS (total mean score 17.2 (SD = 6.1)). The MOCI (Maudsley Obsessive-Compulsive Inventory), which was only used for screening in healthy subjects, showed a very low (subclinical) presence of obsessive-compulsive symptoms with an average score of 6.9 (SD = 3.5).

### Investigation of the experience of time (subjective measure)

For the focus of the study on the dimensions of time, the Bochum Time Questionnaire was used to collect statements on subjective experience of time. Based on 20 items, this questionnaire allows for first-person statements on the experience of time and how the test subjects deal with their own lifetime. The statements were evaluated using a visual analog scale ranging from “Does not apply at all” (corresponding to a value of 1) to “Applies completely” (corresponding to a value of 10). The 20 items in the questionnaire were divided into three categories (knowledge of time = 7 items, experience of time = 7 items, use of time = 6 items), which are based on Karl Jaspers’ [21] dimensions of time. For the evaluation, both the summarized categories as total values and each category (subscale value) were used.

### Investigation of the experience of time (objective measures)

**Fig. 1:**
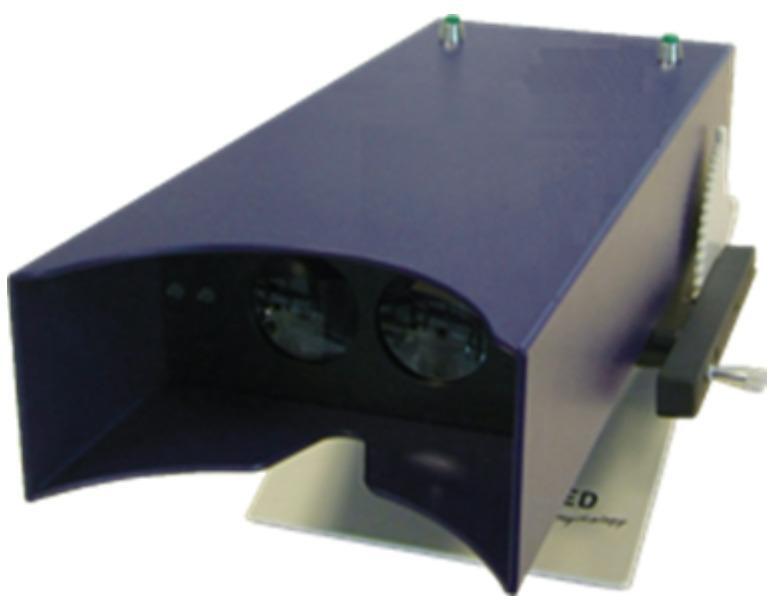
HEPAto-norm™ analyzer for measuring flicker fusion frequency

The following instrumental methods were used to investigate objective time experience, i.e., time perception and motor skills:

The Critical Flicker Fusion Frequency (CFF), also known as the flicker fusion rate, is the frequency at which successive flashes of light are perceived as a continuous light signal. The higher the CFF, the faster we can detect changes in our environment. For example, a fly, whose CFF is approximately 120 Hz, perceives human movements as greatly slowed down. In humans, the CFF ranges between 20 and 90 Hz and depends on physical parameters such as light intensity as well as neural variables. The CFF is considered an indirect measure of the excitation level of the cerebral cortex and is associated with an underlying impairment of executive functions [22]. The flicker fusion frequency was measured using the HEPAto-norm™ analyzer (Fig. 1). The HEPAto-norm™ analyzer consists of headband glasses, a hand-held control device, and a stop button. The headband glasses were held in front of the subject’s eyes and generated an LED light point at a frequency of 60 Hz. The frequency was then continuously reduced to 25 Hz. The test subject indicated when the light signal was no longer perceived as continuous by pressing the stop button. The frequency at which the test subject was able to distinguish individual light signals for the first time was recorded and listed in the “DOWN” measurement series. To generate the second measurement series “UP,” the frequency of the light signals was continuously increased from 25 Hz to 60 Hz. In this case, the test subject pressed the stop button when they first perceived the light signal as constant (flicker fusion frequency). The measurement took place in a room darkened by curtains.

**Fig. 2:**
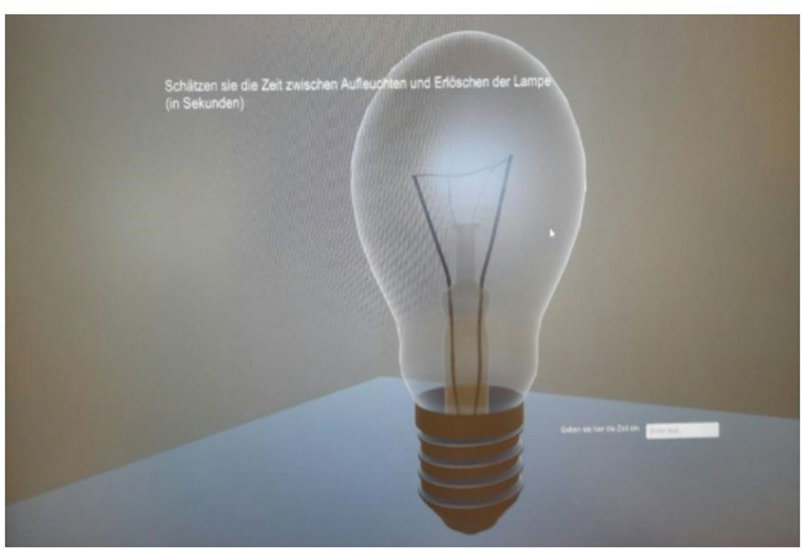
PC-based time measurement “Chronotest” for measuring time estimation

*PC-based time measurement* (“Chronotest,” see Fig. 2) is a computer-controlled test for verbal time estimation and discrimination of time intervals, which is an adaptation of the “Chronotest” [23] (programmed by A. Berns, Göttingen). This test includes a task for verbal time estimation. The patient/test subject is asked to state the duration of a light bulb’s glow in seconds. In addition, as part of a time production task, the light bulb is to be made to glow for a specific (previously specified) period of time by pressing the keyboard. Six runs were performed for each task, with only runs 2 to 6 being recorded and evaluated. The data from the first run was discarded to avoid habituation effects. The duration of the individual runs was determined before the start of the survey and was consistent across all tests. The duration of the runs for verbal time estimation was 5, 1, 13, 2, and 32 s. For time production, time spans of 4, 1, 27, 2, and 14 s were specified.

Finally, the *Vienna Test System* (WTS) from SCHUHFRIED, which allows neuropsychological testing, was used to examine concentration, hand and leg motor skills, and coordination by recording reaction times. The test subject’s task is to react as quickly as possible to sequentially presented visual or.

acoustic stimuli as quickly as possible. Depending on the test form (in this case, the S4/Hanover form was used), the reaction is made by pressing the corresponding keys on the test subject’s keyboard or by pressing the foot keys.

### Statistical analysis

The IBM SPSS 29 program was used for the statistical evaluation of the collected data. The descriptive analysis was performed by calculating frequencies, means, standard deviations, and ranges. The statistical comparisons of the patient and control groups as well as the correlation tests were performed using parametric and non-parametric tests (Chi², Mann-Whitney U test, t-test, Spearman or Pearson correlation coefficients). In addition, due to the large number of characteristics examined here, multivariate covariance analyses were performed, as well as separate multiple linear regression analyses to examine possible influencing factors and their strength on the extent of the target variables in more detail. Due to the characteristics of a pilot study there was no statistical correction procedure as e.g. Bonferroni. Values of p *≤* 0.05 were considered statistically significant and values of p *≤* 0.10 were considered a statistical trend.

## Results

### Descriptive changes in the experience of time

The *objective data* collected in the Chronotest time measurement program were normally distributed for both groups. There were no significant differences between the groups in terms of time estimation or the production of time intervals, although patients tend to overestimate time more than healthy subjects (Table 2). The differences in the measured flicker fusion frequencies between the groups studied here were also not significant. Furthermore, no significant differences in performance were found between the patients and the healthy controls in the context of the various tasks of the Vienna Test System, either in terms of qualitative (accuracy) or quantitative aspects such as reaction times. Only in terms of timely responses did the patients achieve a lower rate (123.3 (SD = 57.1) vs. 156.2 (SD = 61.8)) than the healthy subjects, although this was only a statistical trend with *p* = 0.089. The examination of motor skills using the FFB also showed a statistical trend (*p* = 0.061) in the sense that the patients had more (but not significant) motor impairments than the healthy subjects.

**Table 2:** Time-related parameters

Characteristic (M, SD)	OCD *n* = 20	Healthy *n* = 20	*p*-value*
FB time knowledge	18.2 (11.0)	5.8 (7.6)	p *≤* 0.001
FB time experience	19.9 (11.4)	9.2 (7.7)	*p* = 0.002
FB time management	20.5 (10.8)	4.9 (6.3)	p *≤* 0.001
FB time sum value	58.6 (29.0)	19.9 (19.8)	*p* = 0.001
Flimmer fusion frequency	43.6 (4.0)	44.2 (4.7)	*p* = 0.698; n. s.
Chronotest estimation (MA)	− 1.4 (2.8)	− 0.65 (2.2)	*p* = 0.355; n. s.
Chronotest production (MA)	1.33 (4.1)	− 0.10 (3.0)	*p* = 0.211; n. s
FFB motor skills	74.1 (16.2)	82.4 (10.5)	*p* = 0.061^§^

*M* mean values, *SD* standard deviations, *MA* mean deviation, *n. s.* not significant

*Mann-Whitney U test or t-test

^§^Statistical trend

The *subjective measure* with the time questionnaire revealed that OCD patients achieved significantly higher scores than healthy subjects in terms of greater impairment. This applies to the average scores for the entire questionnaire as well as to the individual categories of time knowledge, time experience, and time management (Table 2).

### Statistical relationships between the parameters

When looking at the correlative relationships of subjective time experience separately for both study groups, significant correlations were found primarily for the patient group, especially with psychometric characteristics. Apart from the correlations listed in Table 3, no further correlations between the characteristics recorded in this study and the experience of time (questionnaire time sum value = FB-ZSW) could be established. A particularly interesting positive significant correlation (*r* = 0.641; *p* = 0.002) was found between the severity of compulsive behaviors (Y-BOCS behaviors) and the score achieved in relation to time management (assessed using the time questionnaire). Correlation coefficients between time management and Y-BOCS remained significant after partialing out BDI (*r* = 0.48; *p* = 0.05) as well as FBB motor skills (*r* = 0.62; *p* = 0.0025) and STAI (*r* = 0.60; *p* = 0.006). Concerning time knowledge the correlation coefficients also remained significant after partialing out FFB motor skills (*r* = 0.54; *p* = 0.02) and STAi (*r* = 0.53; *p* = 0.02), but not BDI (*r* = 0.16; n.s.)

**Table 3:** Correlations between subjective time experience (FB time sum value = ZSW) and the other characteristics examined here

Parameters	FB-ZSW of OCD patients	FB-ZSW of healthy individuals
Age	*r* = 0.508; *p* = 0.022	*r* = 0.096; n. s. (*p* = 0.688)
Partnership	*r* = − 0.708; p *≤* 0.001	*r* = − 0.585; *p* = 0.007
CGI	*r* = 0.685; p *≤* 0.001	/
PSP	*r* = − 0.611; *p* = 0.004	*r* = − 0.372; n. s. (*p* = 0.106)
BDI-II	*r* = 0.527; *p* = 0.017	*r* = 0.470; *p* = 0.037
Y-BOCS actions	*r* = 0.500; *p* = 0.025	/
Y-BOCS total	*r* = 0.523; *p* = 0.018	/

## Discussion

In this exploratory cross-sectional study on subjective and objective measures of time perception in patients with obsessive-compulsive disorder and in comparison to healthy subjects, instrumental measurement methods were used for the first time under standardized conditions to collect potentially underlying neurobiological parameters. Significant changes in the subjective perception of time were found in patients with obsessive-compulsive disorder compared to healthy control subjects. The patient group showed poorer scores for subjective time knowledge, time experience, and time management. This overall change in time experience showed a clear correlation in patients (but also in healthy subjects) with the severity of depressive symptoms as self-assessed using the BDI. In patients with obsessive-compulsive disorder, subjective measures of time were also closely associated with the severity of obsessive-compulsive symptoms. In particular, the extent of compulsive behaviors was associated with a significant impairment in subjective time management. It should be emphasized that in this study, subjective time perception was found to be more clearly and unambiguously disturbed in the patient group than the time measures that can be objectively assessed using flicker frequency and chronotests, such as time perception, time estimation, and time production. This corresponds to previous findings in the literature [16–19, 24] that changes in objective time perception are generally less pronounced than changes in subjective time experience in classic psychiatric disorders (with the exception of brain disorders) such as depression or schizophrenia. In our previous study with a similar study design and with 34 depressed patients, 31 patients with a schizophrenic disorder, and 33 healthy controls [19], we also found significant differences between the three groups in terms of subjective experience of time. However, it was interesting to note that schizophrenic patients in particular also showed conspicuous values in time estimation tasks (estimation of a given time duration) and in flicker fusion frequency as an expression of disturbed time perception, consistent with the concept of schizophrenia as a brain development disorder with microfunctional changes and correspondingly stronger imaging findings [25], while the depressed patients were more similar to healthy individuals in this respect.

The extent to which primary compulsive slowness due to motor impairments may also be a factor cannot be conclusively assessed on the basis of our study. However, based on the sparse literature on compulsive slowness, the phenomenon appears to be more strongly related to psychodynamic aspects [26] and not only in the context of repetitive movement disorders [6, 13]. On the other hand, previous studies [7, 27] have suggested that both fine motor skills (“neurological soft signs,” NSS) and gross motor skills are frequently impaired in OCD patients as a result of basal ganglia dysfunction, which leads to time perception of time in OCD as compulsively slow. A recent review and meta-analysis of a total of 27 studies concludes, however, that NSS are by no means consistent findings in OCD patients and that, for example, patients with schizophrenic disorders are much more severely affected than OCD patients [28]. Furthermore, Ekinci and Ekinci [29] state that patients with compulsive symptoms should be nosologically differentiated from OCD patients with additional tics, as the latter mainly exhibit NSS.

Once again, the heterogeneity of obsessive-compulsive disorders is evident not only on a psychopathological-phenomenological level, but also on a motor-neurological level. The examination of motor skills, both fine and gross motor skills, urgently requires more objective but also practicable assessment and examination instruments for differentiated assessment, which could also contribute to a more concrete classification of the symptoms of compulsive slowness concerning time perception in this disorder. Perhaps, on a more neurobiological and evolutionary perspective on OCD, the role of basal ganglia in regard to habits and ritual behavior could provide a different perspective. Our finding that increased compulsive behavior is associated with poorer time management cannot by clarified in terms of causality. It is however understandable that a person who is preoccupied with compulsive behaviors does not have time for other everyday activities or is unable to manage their time effectively, resulting in slowness because certain tasks could not be completed. This is a broad clinical observation in many cases. .

Our exploratory study, designed as a pilot study, has a number of limitations. One significant limiting factor is certainly the existing psychopharmacotherapy of the patient group, as all patients examined here were medicated for OC symptoms and were of moderate severity. In addition, it should not be forgotten that the patients also exhibited additional depressive symptoms that should not be underestimated. Furthermore, the group size may not have been sufficient for some aspects of the study, such as the time measurement program and further diagnostic testing. In future studies, increasing the number of cases could lead to more meaningful results. The time questionnaire was compiled independently based on international phenomenological templates and had not been validated in a previous study. However, the clear differences compared to the healthy subjects showed that it was possible to achieve meaningful and valid results with it. It should certainly be emphasized that the subjective measures, i.e., those available only to the test subjects, yielded sustainable results in this study. In the spirit of an exploratory pilot study, no Bonferroni correction was performed. It is possible that larger effects could be measured by defining the inclusion and exclusion criteria more strictly. No subgroup differentiation could be made among the patients examined for OCD. This would require a much larger number of cases, as patients with more pronounced obsessive thoughts and few or no compulsive actions may have a different disorder profile with regard to “time processing” than patients who mainly despair of the compulsive actions in their time performance. Furthermore, only one aspect of motor function was assessed here, and only self-assessed motor performance was recorded using the FFB Motor Questionnaire, so that no differentiated statements can be made about the actual motor abnormalities, both fine and gross motor.

The aim of the pilot study conducted here was to use heterogeneous methodology to gain an overview of different dimensions of time experiences in patients with obsessive-compulsive disorder. Subsequent studies will now have the opportunity to examine the individual aspects of time experience in more detail. Among other things, the subjective experience of time could be examined more closely in an even more comprehensive self-assessment questionnaire. This could reinforce the correlations already found in this study and possibly provide a basis for giving greater consideration to the experience of time as a parameter in psychopathological assessment and in the subsequent diagnosis of psychiatric disorders. A more comprehensive understanding of the experience of time in various psychiatric disorders, such as obsessive-compulsive disorder, depression, or schizophrenia, would allow for a better understanding of these disorders and possibly their subgroups. The experience of time is an essential dimension of the psyche and our relationship to the world, and it is surprising that earlier attempts (see, for example, Jaspers 1948/1973) have not led to this being established as an important psychopathological dimension in everyday psychiatric work, although today there is a justified prospect of substantiating such a category neurobiologically with findings from modern methods ranging from EEG to high-resolution MRI imaging.

## Data Availability

Data are available on request.
